# Diffusion of an e-learning programme among Danish General Practitioners: A nation-wide prospective survey

**DOI:** 10.1186/1471-2296-9-24

**Published:** 2008-04-25

**Authors:** Frans Boch Waldorff, Annette Plesner Steenstrup, Bente Nielsen, Jens Rubak, Flemming Bro

**Affiliations:** 1 Research Unit and Department of General Practice, Institute of Public Health, University of Copenhagen, Denmark; 2 Memory Disorders Research Unit, The Neuroscience Centre, Rigshospitalet, Copenhagen University Hospital, Denmark; 3 Danish Medical Association, Department of Education, Copenhagen, Denmark; 4 Institute and Research Unit for General Practice, University of Aarhus, Denmark

## Abstract

**Background:**

We were unable to identify studies that have considered the diffusion of an e-learning programme among a large population of general practitioners. The aim of this study was to investigate the uptake of an e-learning programme introduced to General Practitioners as part of a nation-wide disseminated dementia guideline.

**Methods:**

A prospective study among all 3632 Danish GPs. The GPs were followed from the launching of the e-learning programme in November 2006 and 6 months forward. Main outcome measures: Use of the e-learning programme. A logistic regression model (GEE) was used to identify predictors for use of the e-learning programme.

**Results:**

In the study period, a total of 192 different GPs (5.3%) were identified as users, and 17% (32) had at least one re-logon. Among responders at first login most have learnt about the e-learning programme from written material (41%) or from the internet (44%). A total of 94% of the users described their ability of conducting a diagnostic evaluation as good or excellent. Most of the respondents used the e-learning programme due to general interest (90%). Predictors for using the e-learning programme were Males (OR = 1.4, 95% CI 1.1; 2.0) and members of Danish College of General Practice (OR = 2.2, 95% CI 1.5; 3.1), whereas age, experience and working place did not seem to be influential.

**Conclusion:**

Only few Danish GPs used the e-learning programme in the first 6 months after the launching. Those using it were more often males and members of Danish College of General Practice. Based on this study we conclude, that an active implementation is needed, also when considering electronic formats of CME like e-learning.

**Trial Registration:**

ClinicalTrials.gov Identifier: NCT00392483.

## Background

Internet-based education offers the possibility for professional education and training of physicians and refers to the use of internet technologies to deliver a broad array of solutions that enhance knowledge and performance for the user [1]. In 2001, only 2.7% of physicians in the United States used the Internet for Continuous Medical Education (CME) [2]. However, this figure has recently increased to an estimated 31% and is expected to increase in the coming years [3].

The literature in medical education indicates that internet-based programmes are as effective in increasing participant knowledge as traditional formats [4-6]. In the non-medical literature it has been demonstrated that e-learning can result in significant cost-savings, sometimes as much as 50%, compared with traditional instructor-led learning [7]. Studies in both medical and non-medical literature have consistently demonstrated a high learner satisfaction with e-learning and the satisfaction rates increase with e-learning compared to traditional learning, along with perceived ease of use and access, navigation, interactivity, and user-friendly interface design [7].

Clinical guidelines, in the form of systematically developed statements to assist physician-patient decisions on appropriate health care for specific circumstances are produced in many countries [8]. However, the dissemination of guidelines alone rarely brings about improvements in clinical practice [9] and even an implementation of guidelines may not change clinical practice [10,11]. We were unable to identify studies that have considered the diffusion of an e-learning programme among general practitioners. *Diffusion of Innovations*, theorized that innovations would spread through society in an s-curve, as early adopters select the technology first, followed by the majority, until a technology or innovation is common. Due to the assumption that the use of e-learning programmes, as a source to CME, will increase in the future we found it of interest to study the diffusion of an e-learning programme.

The aim of this study was to investigate the uptake of an e-learning programme introduced to General Practitioners as part of a nation wide disseminated dementia guideline.

## Methods

The study was conducted as nation-wide prospective survey in Denmark among all Danish GPs from November 3rd 2006–May 3rd 2007.

### Participants

The GPs were identified by the database of the Danish Medical Association (DMA) on May 3rd 2007. Characteristics of Danish GPs are presented in Table 1.

**Table 1:** Characteristics of Danish GPs (N = 3.632)

Average Age (SD)	54.2 (7.6)
Proportion of females	35.9%
Members of the Danish College of General Practice	65.3%
Years since graduation (SD)	25.2 (8.2)
Working place*	
Municipalities with more than 50.000 inhabitants	41.6% (1.511)
Municipalities with cities from 20.000–50.000 inhabitants	38.5% (1399)
Municipalities up to 20.000 inhabitants	19.9% (722)

*Based on data from January 1.st 2005.

### The guideline and the e-learning programme

The Dementia Guideline was developed by the Danish College of General Practitioners (DSAM) and mailed to all Danish General Practitioners (GPs) in October 2006 [12]. The dissemination strategy was chosen by DSAM and the ELP producer jointly. The e-learning programme (ELP) was mentioned in the guideline in a separate section and was launched in the beginning of November 2006. The ELP followed the recommendation of the Guideline and consisted of five sections: Suspicion of dementia, identification, diagnostics, evaluation and follow-up. The ELP was based on interactive parts: using slides with audio, video-cases and self-study parts. The estimated time to complete the ELP was 90 minutes and the producers did not incorporate an evaluation of performance. All GPs had free access to the ELP from the homepages of DSAM as well as DMA by a unique username and password. At the launching the ELP was promoted at the homepages of the DMA as well as DSAM, the ELP was mentioned in the weekly journal of DMA (Ugeskrift for Læger) as well as the journal of DSAM (Practicus). Furthermore, DSAM sent one email to all members with a stated email address. The Guideline and the ELP was financially supported by the Danish Ministry of Health.

### Data collection

Data was collected at the logon by Internet based pop up questionnaires; log files, as well as from databases at the DMA. The data was provided with a constructed identification code. Thus, data on the individual GP identity was kept anonymous for the authors.

### Internet based pop up questionnaires

At the introduction page all users were informed that they participated in an evaluation of the ELP and were encouraged to complete the questionnaires. However, participants could choose to start the ELP without completing the questionnaires. There were two questionnaires both of which were tested for face validity before the launch of the ELP by letting five GPs fill out the items and adjusting them according to the evaluation. The first questionnaire should be completed at the first logon. This questionnaire consisted of 7 items. All items are presented in Table 2.

**Table 2:** Statements from GPs using the e-learning programme for the first time (N = 125)

	% (n)
Item 1. Where have you heard about the e-learning programme?*	
Written material	42 (52)
On the internet	44 (55)
Recommended by peer	6 (7)
Other	28 (35)
Item 2. *How would you describe your ability to perform diagnostic evaluation of dementia? *(n = 124)	
Excellent	1 (1)
Good	40 (50)
Fair	53 (66)
Less good	5 (6)
Bad	1 (1)
Item 3. *Have you read the latest version of the Dementia guideline? # *(n = 122)	
Yes	62 (76)
No	38 (46)
Item 4. *Why did you use the e-learning program this time? # *(n = 124)	
General interest	90 (112)
Problem with a specific diagnostic evaluation	2 (3)
Other	7 (9)
Item 5. *Have you Internet access in your clinical office? *(n = 124)	
Yes	98(122)
No	2 (2)
Item 6. *How often do you use the Internet when you are working? *(n = 120)	
Every day	88 (105)
Every week	9 (11)
less than every week	3 (4)
Item 7. *How many persons are around you now when you are using this e-learning programme? *(n = 124)	
Only me	94 (117)
One beside me	2 (3)
Two beside me	3 (4)

*participants could tick out more than one items

#Items repeated in questionnaire two but not presented in Table 3

The second questionnaire should be completed at subsequent logons. This questionnaire consisted of 5 items. Item one and two were repeated from Questionnaire one (Please refer to Table 2). The remaining three items are presented in Table 3.

**Table 3:** Statements from GPs at subsequent log-on(s) at the e-learning programme (n = 60)*

	% (n)
Item 1. *How would you describe the ability to navigate in the e-learning programme? *(n = 60)	
Extremely easy/easy*	58 (35)
Fair	37 (22)
Difficult/extremely difficult**	5 (3)
Item 2. *Did you find the answer you were trying to find? *(n = 59)	
Yes	68 (40)
No	7 (4)
I don't know	25 (15)
Item 3. *Would you recommend this e-learning programme to colleagues? *(n = 58)	
Yes	86 (50)
No	2 (1)
I don't know	12 (7)

*Based on report(s) from 32 GPs

**Reported pooled, in this table

### Log files

These files were retrieved from a central database by the software provider (Cursum). Each click by a mouse is filed in a log file, which makes it possible to examine the use and time spent on the e-learning programme. When no mouse click was detected for 20 minutes the programme was terminated and no time was calculated. Log files were obtained as plain text.

### Data from Danish Medical Association

Information's regarding: Sex, age, year of graduation from university, working address, type of practice and membership of the Danish College of General Practitioners and logons at the e-learning programme were retrieved from the DMA database.

### Statistics

We used chi-square and t-tests to compare differences between users and non-users. In order to identify predictors for using the e-learning programme the probabilities and corresponding 95% confidence intervals were estimated based on logistic regression analysis using with the GEE methods. Pearson's chi-square was used to evaluate Goodness of Fit for the model. A deviance approximately equal to its degrees of freedom has been suggested as a possible indication of a good model fit.

The following variables were included in the model as predictors: age, gender, membership of the Danish College of General Practitioners and municipality size. Age was dichotomized at 55 years and experience was dichotomised at 15 years. All statistical analyses were performed using SAS, version 9.1, proc Gee (SAS Institute Inc, Cary, NC).

### Ethics

The Scientific Ethical Committee for Copenhagen and Frederiksberg Municipalities evaluated the project. The Danish Data Protection Agency and the Danish College of General Practitioners Study Committee approved the project. The project was registered in ClinicalTrials.gov Identifier: NCT00392483.

## Results

In the study period of 6 months a total of 192 different GPs (5.3%) were identified as users of the e-learning programme, and 17% (32) had at least one re-logon. A total of 125 participants (66%) completed the pop up questionnaire at the first log on (Table 2). Most of the GPs (81%) stated at the first log on that they had gained information about the e-learning programme from one source, whereas the remaining (24) had gained information from two or more different sources. A total of 90% stated that the log on session was due to general interest and 94% were sitting alone when using the e-learning programme. Among those 32 with subsequent logon(s) the perceived ease of use and navigation was in general good (Table 3). Most of the re-logons (65%) were conducted within 24 hours from the first logon. Based on a logistic regression model, predictors for using the ELP were examined. Males (OR = 1.4, 95% CI 1.1; 2.0) and members of DSAM (OR = 2.2, 95% CI 1.5; 3.1) were more likely to use the ELP, whereas age, experience and working place did not seem to be influential (Table 4).

**Table 4:** Predictors for GPs using the e-learning programme in a 6 month period (N = 3632)

	Odds Ratio (95% CI)	p-value
Age under 55	1.2 (0.8;1.8)	0.31
Males	1.4 (1,1;2.0)	0.04
Members of Danish College of General Practice	2.2(1.5; 3.1)	<0.001
Municipalities with more than 20.000 inhabitants	0.7 (0.5;1.1)	0.11
Graduation at University	1.2 (0.7;2)	0.51

## Discussion

To our knowledge, this is the first study to examine the diffusion of an e-learning programme (ELP) in General Practice. Only few GPs used the ELP in the study period. The GPs using the ELP were in general sitting alone and did not have a specific diagnostic problem. Male GPs and Members of Danish College of General Practitioners may be more inclined to use the ELP. It was possible to identify a total of 5.3% of Danish GPs that had at least one log on session within a period of 6 months from launching of the ELP. This figure was lower, than we had expected. One reason may be that the mailed letter did not evoke the GPs attention. Another reason may be that the ELP did not appeal to the GPs or a lack of perceived need for CME in dementia. An active implementation strategy could have included emails with direct link to the ELP or small group CME activities. Some GPs may prefer written material or courses as a tool for learning. The low figure may also be due to the new technology that ELP represent. Trying a new technology may be considered as a barrier [13].

Among the responses from those GPs using the ELP more than once there was a clear indication that they actually found it easy to use, found what they were looking for and would recommend it to colleagues. The fact, that the participants completing the questionnaires in general had a high self-rated competency in diagnostic evaluation of dementia, and were using the programme due to general interest, indicate that the GPs with at least one log on session were specially motivated physicians that may not belong to the target group of this CME activity. Furthermore, members of DSAM were more inclined to log on, which may be explained by the email sent to members by the DSAM. According to the diffusion of innovation we assume that the participant are early adaptors and thus, may not be considered as a representative group of Danish GPs. The finding that the ELP users are more often members of the College of General Practitioners supports this. We can therefore expect that the responses regarding performance (Table 2) may score higher than average Danish GPs.

However, some GPs may have used other ways to gain information than this ELP and that self-reports may overestimate actual performance. We would like to stress, that there are different means of learning and they may change over time, and e-learning is only one of the means. Furthermore, this was the first ELP aimed directly at GPs, and integrated in the dissemination of a guideline, thus ELP has not been established as a possible CME activity in the mind of most GPs. We have not identified other studies reporting the number of persons using an ELP. Most of the participants were sitting alone when using the ELP, even though we think there is a great learning possibility in ELP for groups.

Dementia is a common condition in the elderly, with an estimated prevalence of 7.5% those above 65 years of age [14]. However, previous surveys have indicated that a substantial fraction of patients with cognitive symptoms are not diagnosed by GPs [13], that diagnostic evaluation in general practice is difficult [11,15] and that several barriers for diagnostic evaluation in patients and their caregivers as well as among GPs exists [13]. Improvement in the handling of dementia in general practice faces the same barriers as other implementations initiatives: lack of perceived requirement for change; costs; poorly designed implementation; inadequate technology. However, a precondition for improvements is that the GP knows about dementia and how to diagnose it. This ELP aimed at providing the GP with such knowledge and perhaps is 5% a realistic aim for such a mass strategy and reflect the proportion of GPs who are interested in the clinical area and also find the ELP as a learning method fits their learning profile. Previous studies have only considered targeted strongly motivated groups of professionals [16-22] or internet traffic at a web site [23].

Different barriers may be present in different settings at different times. If the implementation methods are tailored to overcome the obstacles, then change may be more likely to take place. In order to identify the obstacles in this present setting an initial survey could have been initiated before the launching of the ELP. This could possibly have improved the implementation strategy and the subsequent number of users.

In accordance with other studies, we used log-files from the ELP software provider as an outcome measure [21]. This methodology ensures that our data are complete and correct and that the proportion of participants can thus be considered to be accurate. Thus, we believe that this result could be generalised in a Danish setting. Danish general practices were computerized at an early stage and Danish GPs are probably at least as prone to use information technology tools such as ELP as GPs elsewhere.

However, this outcome measure does not evaluate the actual learning progress of the individual physician using the ELP. In our study, we had the advantage of having the possibility to identify users by some basic characteristics due to the possibility of identifying the participants by a unique log-on code and a subsequent merging of the log files with the DMA member database. To secure anonymity for the GPs we received data with a newly constructed participation code, which did not allow us to identify individual GPs. Thus, we do not have any ethical concerns regarding this study. However, we could have increased knowledge about non-users by mailing a questionnaire to a random sample of non-users. We did not consider this, because of the constructed participant code, which did not allow us to identify the individual GP.

## Conclusion

This study demonstrated that only few GPs used an e-learning programme for dementia developed for and by GPs. This was the first ELP introduced to Danish GPs and the user rate may increase in the future, when the GPs become familiar with this technology. The dissemination and promotion of the ELP was planned and implemented independently of this study. However, this study underscores the need to identifying barriers and plan implementation strategies accordingly.

## Competing interests

The study was financially supported by The Danish Ministry of Health. FBW has received research funding from the sponsor.

## Authors' contributions

FBW participated in the design of the study, acquisition of the data, analysis, drafting and revising of the manuscript. FBW, APS, BN and JR participated in the design of the study, interpretation of data and revising the manuscript. All authors read and approved the final manuscript.

## Pre-publication history

The pre-publication history for this paper can be accessed here:



## References

[B1] M R (2001). E-Learning: Strategies for Delivering Knowledge in the Digital Age.

[B2] Brown TT, Proctor SE, Sinkowitz-Cochran RL, Smith TL, Jarvis WR (2001). Physician preferences for continuing medical education with a focus on the topic of antimicrobial resistance: Society for Healthcare Epidemiology of America. Infect Control Hosp Epidemiol.

[B3] A R, JA. MD (2006). Evaluating technology-enhanced continuing medical education.. Med Educ Online.

[B4] Cobb SC (2004). Internet continuing education for health care professionals: an integrative review. J Contin Educ Health Prof.

[B5] Ruiz JG, Mintzer MJ, Leipzig RM (2006). The impact of E-learning in medical education. Acad Med.

[B6] Wutoh R, Boren SA, Balas EA (2004). eLearning: a review of Internet-based continuing medical education. J Contin Educ Health Prof.

[B7] A G, P F, S T and J F (2002). Computer-based instruction. Training & Retraining: A handbook for business, Industry, Goverment and the Military.

[B8] Grimshaw J, Freemantle N, Wallace S, Russell I, Hurwitz B, Watt I, Long A, Sheldon T (1995). Developing and implementing clinical practice guidelines. Qual Health Care.

[B9] Wensing M, van der WT, Grol R (1998). Implementing guidelines and innovations in general practice: which interventions are effective?. Br J Gen Pract.

[B10] Bro F, Waldorff FB (2004). Guidelines--let's take a break and then move forward together!. Scand J Prim Health Care.

[B11] Waldorff FB, Almind G, Makela M, Moller S, Waldemar G (2003). Implementation of a clinical dementia guideline. A controlled study on the effect of a multifaceted strategy. Scand J Prim Health Care.

[B12] (2006). Danish College of General Practitioners. http://www.dsam.dk.

[B13] Waldorff FB, Rishoj S, Waldemar G (2005). Identification and diagnostic evaluation of possible dementia in general practice. A prospective study. Scand J Prim Health Care.

[B14] Lobo A, Launer LJ, Fratiglioni L, Andersen K, Di Carlo A, Breteler MM, Copeland JR, Dartigues JF, Jagger C, Martinez-Lage J, Soininen H, Hofman A (2000). Prevalence of dementia and major subtypes in Europe: A collaborative study of population-based cohorts. Neurologic Diseases in the Elderly Research Group. Neurology.

[B15] Renshaw J, Scurfield P, Cloke L, Orrell M (2001). General practitioners' views on the early diagnosis of dementia. Br J Gen Pract.

[B16] Schilling K, Wiecha J, Polineni D, Khalil S (2006). An interactive web-based curriculum on evidence-based medicine: design and effectiveness. Fam Med.

[B17] Wehrs VH, Pfafflin M, May TW (2007). E-learning courses in epilepsy--concept, evaluation, and experience with the e-learning course "genetics of epilepsies". Epilepsia.

[B18] Vandeweerd JM, Davies JC, Pinchbeck GL, Cotton JC (2007). Teaching veterinary radiography by e-learning versus structured tutorial: a randomized, single-blinded controlled trial. J Vet Med Educ.

[B19] Whitfield L (2007). Back to the new school. Health Serv J.

[B20] Lewis PA, Price S (2007). Distance education and the integration of E-learning in a graduate program. J Contin Educ Nurs.

[B21] Filippucci E, Meenagh G, Ciapetti A, Iagnocco A, Taggart A, Grassi W (2007). E-learning in ultrasonography: a web-based approach. Ann Rheum Dis.

[B22] Ridgway PF, Sheikh A, Sweeney KJ, Evoy D, McDermott E, Felle P, Hill AD, O'Higgins NJ (2007). Surgical e-learning: validation of multimedia web-based lectures. Med Educ.

[B23] Sparacia G, Cannizzaro F, D'Alessandro DM, D'Alessandro MP, Caruso G, Lagalla R (2007). Initial experiences in radiology e-learning. Radiographics.

